# Genotype–Phenotype Correlations in 293 Russian Patients with Causal Fabry Disease Variants

**DOI:** 10.3390/genes14112016

**Published:** 2023-10-28

**Authors:** Kirill Savostyanov, Alexander Pushkov, Ilya Zhanin, Natalya Mazanova, Alexander Pakhomov, Elena Trufanova, Alina Alexeeva, Dmitry Sladkov, Ludmila Kuzenkova, Aliy Asanov, Andrey Fisenko

**Affiliations:** 1 FSAI National Medical Research Center for Children’s Health of the Russian Federation Ministry of Health, Moscow 119991, Russia; pushkovgenetika@gmail.com (A.P.); ilya_zhanin@outlook.com (I.Z.); genelab@nczd.ru (N.M.); al_pachomov@mail.ru (A.P.); quark-gluon@mail.ru (E.T.); alinalaboratoria@gmail.com (A.A.); dmitry.sladkov93@gmail.com (D.S.); kuzenkova@nczd.ru (L.K.); director@nczd.ru (A.F.); 2 Federal State Autonomous Educational Institution of Higher Education I.M. Sechenov First Moscow State Medical University of the Ministry of Health of the Russian Federation (Sechenov University), Moscow 119991, Russia; aliy@rambler.ru

**Keywords:** Fabry disease, selective screening, hypertrophic cardiomyopathy, NGS, α-gal A, lyso-Gb3, *GLA*

## Abstract

Background: Fabry disease (FD) is a rare hereditary multisystem disease caused by variants of the *GLA* gene. Determination of *GLA* gene variants and identification of genotype–phenotype correlations allow us to explain the features of FD associated with predominant damage of one or another system, both in the classical and atypical forms of FD, as well as in cases with late manifestation and involvement of one of the systems. Methods: The study included 293 Russian patients with pathogenic variants of the *GLA* gene, which were identified as a result of various selective screening programs. Screening was carried out for 48,428 high-risk patients using a two-step diagnostic algorithm, including the determination of the concentration of the biomarker lyso-Gb3 as a first-tier test. Screening of atypical FD among patients with HCM was carried out via high-throughput sequencing in another 2427 patients. Results: 102 (0.20%) cases of FD were identified among unrelated patients as a result of the study of 50,855 patients. Molecular genetic testing allowed us to reveal the spectrum and frequencies of 104 different pathogenic variants of the *GLA* gene in 293 examined patients from 133 families. The spectrum and frequencies of clinical manifestations in patients with FD, including 20 pediatric patients, were described. Correlations between the concentration of the lyso-Gb3 biomarker and the type of pathogenic variants of the *GLA* gene have been established. Variants identified in patients with early stroke were described, and the association of certain variants with the development of stroke was established. Conclusions: The results of a large-scale selective FD screening, as well as clinical and molecular genetic features, in a cohort of 293 Russian patients with FD are described.

## 1. Introduction

Fabry disease (FD) is a rare hereditary multi-system disease caused by variants of the *GLA* gene, leading to reduced α-galactosidase A activity and, as a result, to the accumulation of toxic glycosphingolipids in various organs and tissues [1]. The average incidence of FD is 1 case per 40,000 live-born male infants [2]. The FD incidence among dialysis patients is 1 case per 1000 male patients [3]; among patients with idiopathic cardiomyopathy, it is 1 case per 30 male patients [4]; among patients with cryptogenic strokes, it is 1 case per 20 male patients and 1 case per 40 female patients [5]. In total, FD occurs in 8.8% of cases among patients with metabolic diseases [6] and is the second most common lysosomal storage disease (LSD) [7].

Pathogenesis of FD is based on pathogenic changes in the nucleotide sequence of the *GLA* gene located in the Xq22 chromosomal region. The *GLA* gene is 12 kb long and includes seven exons [8]. The pathogenic effect of the same variants can lead to different phenotypes in different carriers due to random X-inactivation and lack of cross-correction [9], ranging from an asymptomatic disease course or its mild form to a severe course comparable to the disease in men [10]. There are a few women (with variants) who do not exhibit any manifestations of FD unlike other X-linked diseases, such as Hunter’s disease [11]. Moreover, only 70% of women with variants are clinically diagnosed [12].

Insufficient enzyme activity of α-galactosidase A leads to the accumulation of glycosphingolipids, and in particular, globotriaosylceramide (Gb3), primarily in cell lysosomes of small blood vessel walls, spinal and autonomic ganglia, renal glomeruli, tubular epithelial cells, and cardiomyocytes. This leads to systemic disorders characterized by chronic pain and acroparesthesia, hearing loss, hypohidrosis, gastrointestinal disorders, corneal opacity, angiokeratomas, progressive renal failure, cardiomyopathy, left ventricular hypertrophy, cardiac valve dysfunction, and stroke [13]. Gb3 and its isoforms have been detected in FD patients’ blood since the beginning of the 2000s [14], but only in 2008, Aerts et al. managed to describe the significant accumulation of deacylated Gb3 metabolite in lysosomes-globotriaosylceramide (lyso-Gb3) [15]. Lyso-Gb3 has shown a higher sensitivity and a reliable correlation with the FD phenotype compared to Gb3 [16]. Previous studies have allowed the clarification of some mechanisms of FD pathogenesis, including incomplete penetrance in women, and also made lyso-Gb3 a reliable biomarker for FD. The measurement of its levels helps to establish diagnosis even in the most controversial cases, as well as to make timely decision on enzyme replacement therapy (ERT) or chaperone therapy administration at laboratory diagnosis and before the first clinical manifestations [17]. Moreover, the measurement of accumulating glycosphingolipids’ levels allows us to monitor the pathogenetic therapy efficacy [15].

Mehta et al. have used data of 1453 patients with FD from 19 countries in their study. They reported that neurological signs were described in 75% of men and 61% of women, renal failure in 19% of men and 3% of women, cardiovascular manifestations in 60% of men and 50% of women, thereby, left ventricular hypertrophy was identified in 40% of men and 24% of women, and stroke in 9% of men and 5% of women. Moreover, it was mentioned that the most common cause of death in FD patients after 2001 was cardiovascular complications, while before 2001, it was renal failure in men and brain vascular diseases in women. This indicates success in combating kidney diseases in FD patients in recent years [18].

The diversity of FD clinical manifestations, the age of onset, and the disease course are determined by many etiological factors of pathogenesis. However, the fundamental factor that triggers the pathological process is the changes in reference to the *GLA* gene. Analysis of *GLA* gene variants and attempts to identify genotype–phenotype correlations help to explain the FD features associated with the predominant damage of a particular system both in the classical and atypical (with late onset or with one system involvement) forms of FD [19]. A total of 45% of variants, among pathogenic variants of the *GLA* gene described by 2003 and leading to classical FD, were nonsense variants, so these variants led to premature translation arrest [20].

Almost all FD complications are nonspecific and clinically similar to other disorders that occur in the general population. This makes clinical diagnosis of FD much more complicated [12]. Moreover, timely diagnosis of FD is an important challenge of modern medicine due to the fact that there is a successful ERT available for FD treatment. ERT with intravenous infusions of α-galactosidase A gradually reduces the level of lyso-Gb3 in blood plasma. It clears lysosomal inclusions from vascular endothelial cells, while its effect on other tissues is not so obvious [21]. Thus, early ERT or chaperone therapy initiation is considered as the most effective therapeutic strategy, especially as some complications are nonreversible despite the concentration of administered intravenous drugs [22].

## 2. Materials and Methods

This study was conducted in accordance with the ethical principles stated in the most recent version of the Declaration of Helsinki and the applicable guidelines on good clinical practice to ensure the greatest protection of individuals. It was also approved by the Ethics Committee of National Medical Research Center for Children’s Health, Moscow, Russian Federation (Protocol No. 13, dated 20 December 2012).

The study included patients referred to our institution for diagnosis from various selective screening programs from November 2013 to December 2022.

### 2.1. Selective Screening of Classical FD

**Figure A1:**
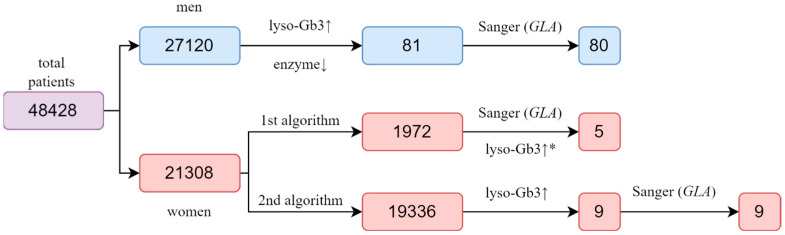
Algorithm of selective screening of classical FD. Sanger sequencing was performed for coding and adjacent intronic regions of the *GLA* gene. *—measured retrospectively.

**Figure A2:**
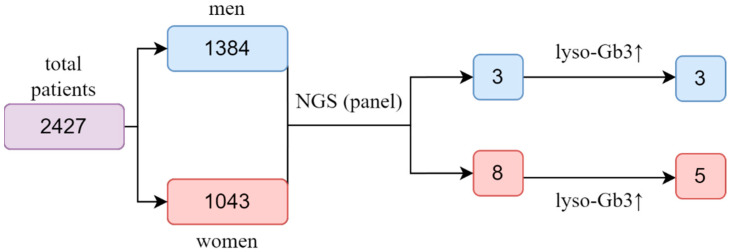
Algorithm of selective screening of atypical FD among patients with HCM. NGS was performed using the custom NGS panel containing target regions of 17 genes: *ACTC1*, *DES*, *FLNC*, *TPM1*, *TTR*, *TNNI3*, *GLA*, *LAMP2*, *MYH7*, *TNNC1*, *TNNT2*, *MYBPC3*, *MYL2*, *MYL3*, *PTPN11*, *PLN*, and *PRKAG2*.

Screening of classical FD was performed with two algorithms (Figure A1 and Figure A2).

Initially, the pilot screening was limited by the sample (n = 1972) of female patients aged 19 to 76 years, and the first-tier test involved the sequencing of the coding and adjacent intronic regions of the *GLA* gene. At the same time, we measured the concentration of lyso-Gb3 in all these women retrospectively.

Next, a two-step diagnostic algorithm was used for men and women. Women (n = 19,336) underwent the evaluation of lyso-Gb3 levels in blood via HPLC-MS/MS at the first stage according to another diagnostic algorithm. Molecular genetic testing was performed via direct automatic sequencing, if the biomarker level was high (cut-off point: 1.91 ng/mL). In total, the lyso-Gb3 levels were measured in 21,308 women during screening. All men enrolled in the study (n = 27,120) aged 6 to 68 years were assessed for their α-galactosidase A activity via MS/MS and lyso-Gb3 levels in dry blood spots via HPLC-MS/MS. Lyso-Gb3 levels were evaluated retrospectively in 3725 (13.5%) patients.

All patients (n = 48,428) examined according to the described screening algorithms included 32,447 (67%) patients admitted from dialysis centers and nephrology departments of specialized hospitals, 11,622 (24%) from neurological departments, and 4329 (9%) from outpatient visits of various outpatient departments. There were 942 children aged 2 to 18 years among the total number of patients examined.

Blood spots dried on filter paper were used as biological material. The α-galactosidase A activity was measured via Bruker Maximis Impact tandem mass spectrometer (Germany, Mannheim) with positive ionization in electrospray with MS-MS. The lyso-Gb3 levels were measured via Bruker Maximis Impact tandem mass spectrometer with positive ionization in electrospray with HPLC-MS/MS.

### 2.2. Selective Screening of Patients with HCM

The study included 2427 patients (1384 males and 1043 females) with a median age of 50 years, who were diagnosed with HCM according to current clinical guidelines. All these patients were referred from cardiological profile hospitals. In the first stage of screening, all patients underwent molecular genetic testing (NGS method) of target regions. These regions included the coding sequences of 17 genes and variants that can lead to the development of HCM. Lysosomal globotriaosylsphingosine (lyso-Gb3) concentration and α-galactosidase A (α-gal A) enzyme activity were measured in the second stage of screening to reveal pathogenic or likely pathogenic variants of the *GLA* gene (NM_000169.3).

### 2.3. Genomic DNA Extraction

Genomic DNA was extracted from blood via DNA Blood Mini Kit, QIAGEN (Germany, Hilden), according to the protocol recommended by the manufacturer. The phenol–chloroform extraction technique was used when working with buccal smears and dried blood spots. The quality and quantity of DNA were estimated spectrophotometrically via NanoPhotometer N60 (Implen, Germany, Munich) and with the Qubit dsDNA HS Assay Kit for the Qubit 3.0 fluorometer (Invitrogen, USA, Waltham).

### 2.4. Sanger Sequencing

Sanger sequencing was performed via the BigDye^®^ Terminator v3.1 Cycle Sequencing Kit (Thermo Fisher Scientific, USA, Waltham) in accordance with the manufacturer’s protocols and guidelines. Amplification was performed on Bio-Rad T100 (Bio-Rad, USA, Santa Rosa) and ProFlex (Thermo Fisher Scientific, USA, Waltham) thermocyclers. Capillary electrophoresis was performed on ABI 3500XL automated DNA sequencer (Thermo Fisher Scientific, USA, Waltham). The obtained sequences were compared with RefSeqGene reference sequences from the National Center for Biotechnology Information’s database.

### 2.5. Next-Generation Sequencing

The target regions of the *ACTC1*, *DES*, *FLNC*, *TPM1*, *TTR*, *TNNI3*, *GLA*, *LAMP2*, *MYH7*, *TNNC1*, *TNNT2*, *MYBPC3*, *MYL2*, *MYL3*, *PTPN11*, *PLN*, and *PRKAG2* genes were analyzed.

Hybridization probes for the NGS panel were designed via the Hyper Design Tool (Roche). They cover all coding and splice regions of the genes listed above and other regions of the described pathogenic variants within introns, such as *GLA* gene region containing pathogenic variant rs199473684. The size of the panel was ~45 kbp.

Libraries for NGS were prepared using a KAPA HyperPlus Kit (Roche, USA, Indianapolis) according to the manufacturer’s protocol. The DNA fragmentation time was 15 min to achieve an average fragment length of 350 bp. Target enrichment was carried out using KAPA HyperCap hybridization probes (Roche, USA, Indianapolis). Massive parallel sequencing was performed on the MiSeq platform (Illumina, USA, San Diego) with V2 chemistry (500 cycles, paired-end reads). On average, in every run, 31.5 million reads were obtained, of which, 88% had a Phred score higher than Q30. Overall, 99% of all target regions were covered at least 15 times, and the mean read depth was 150 times.

### 2.6. Bioinformatic Analysis

Bioinformatic analysis was carried out according to the guidelines of Genome Analysis Toolkit Best Practices (https://gatk.broadinstitute.org/hc/en-us, accessed on 20 September 2023). Briefly, raw reads were trimmed using Trimmomatic (version 0.39). Then, sequence alignment was performed with Burrows–Wheeler Aligner (version 0.7.17) using GRCh37 genome assembly as a reference. Next, duplicate reads were marked using Picard tools, and base quality score recalibration (BQSR) was performed. Then, genetic variations (SNPs and indels) were identified with GATK HaplotypeCaller (version 4.1.2). Next, gene annotation was performed with an in-house script to annotate variations present in ClinVar, OMIM, and HGMD databases. The pathogenicity of variants not previously described was determined using the Alamut Visual Plus (version 1.7.2) with the built-in software modules SIFT, PolyPhen HDIV, PolyPhen HVAR, Mutation Taster, FATHMM, CADD13, DANN, M-CAP, and REVEL, as well as using the ACMG manual. Finally, a filtering process removed variations outside targeted sequences, with a population frequency > 0.5% (gnomAD v2.1.1).

All genetic frameshift variants as well as variants that prematurely interrupt the synthesis of encoded proteins (nonsense) were considered as quantitative variants.

## 3. Results

FD screenings unique for the Russian Federation in terms of the number of performed studies that covered in total 50,846 (28,504 men and 22,342 women) patients from high-risk groups, undergoing hemodialysis therapy [23], patients from neurological and cardiology departments [24], and pediatric patients as well [25] can be considered as examples of selective screening.

Reduced enzyme activity was detected in 81 (0.34%) men out of 27,120 after selective screening. Increased levels of the lyso-Gb3 biomarker were identified in 80 of them. The laboratory diagnosis was confirmed using molecular genetic testing: 80 patients had hemizygous variants of the *GLA* gene. It should be noted that one false-negative and two false-positive results were detected in the determination of α-galactosidase A activity. False-negative enzymatic activity was identified in a man with a lyso-Gb3 level of 8.58 ng/mL and the *c.758T>C* (*p.I253T*) variant of the *GLA* gene described earlier in patients with atypical FD [26]. However, false-positive values of enzymatic activity were identified in two men with a normal level of lyso-Gb3 and with *c.427G>A* (*p.A143T*) and *c.937G>T* (*p.D313Y*) variants of the *GLA* gene with conflicting pathogenicity.

Screening on atypical FD identified three more men with pathogenic variants of the *GLA* gene, reduced activity of α-galactosidase A, and an increased concentration of the lyso-Gb3 biomarker. Two unrelated men had the pathogenic variant *c.902G>A* (*p.R301Q*), described earlier in the international HGMD database of patients with FD, including those exhibiting an atypical disease form, while the third had the pathogenic variant *c.644A>G* (*p.N215S*), described earlier in the international HGMD database in only patients with FD.

Molecular genetic testing was performed (the sequencing of coding and adjacent intronic regions of the *GLA* gene) at the first stage to diagnose women according to the screening initial algorithm. The lyso-Gb3 levels were measured at the next stage, as well as retrospectively.

Following this screening, the change in the reference base sequence of the *GLA* gene was identified in 24 women, while increased lyso-Gb3 levels were identified in only four of them. A total of 19 women with normal lyso-Gb3 levels had nucleotide variants described earlier as variants with conflicting pathogenicity and exhibited a slight decrease in enzymatic activity: *c.247G>A* (*p.N83D*), *c.376A>G* (*p.S126G*), and *c.937G>T* (*p.D313Y*) [7,27]. Moreover, it should be noted that one woman with FD and the pathogenic variant c.1211G>A (*p.R404K*) of the *GLA* gene [28] exhibited a false-negative lyso-Gb3 level, and it was 0.80 ng/mL. Thus, five female patients were identified as a result of this screening algorithm for women with suspected FD, with a detectability of 0.25%.

Another screening algorithm utilized lyso-Gb3 level measurement as the primary diagnostic step for women, while molecular genetic testing was performed only as a confirmatory method. In total, lyso-Gb3 levels were measured in 19,336 women during this screening. As a result, nine more women with increased biomarker levels and changes in the reference base sequence of the *GLA* gene were identified. The detectability in this case was much lower and amounted to 0.042%. Such a low percentage may indicate that, within this screening, women with atypical FD with minimal clinical signs and normal lyso-Gb3 levels could be missed, while only women with the classical form would be identified.

An additional screening of 1043 women with cardiomyopathy identified eight more women with changes in the reference base sequence of the *GLA* gene. Five of them had increased lyso-Gb3 levels, while three women with HCM had biomarker levels within their normal values. First, a novel variant *c.971T>G* (*p.L324W*) was identified, second, a variant with contradictory pathogenicity *c.427G>A* (*p.A143T*) was identified, and third, we found a deletion *c.640-794_640-791del*, located deep in the intron close to the pathogenic variant *c.640-801G>A*, described in patients with late form of FB with HCM [29].

Thus, as a result of selective screening of 50,846 patients from high-risk groups, we identified 102 (0.20%) cases of FD among unrelated patients. This result corresponds well with similar international studies [30].

In contrast to screenings performed in men, the lyso-Gb3 biomarker does not have 100% sensitivity and specificity for FD diagnosis as observed in women. The most preferable algorithm is one that uses molecular genetic testing at the first stage and measurement of lyso-Gb3 levels at the second stage. However, such an algorithm is significantly more expensive and cannot be widely used without proper financial support.

**Figure 1:**
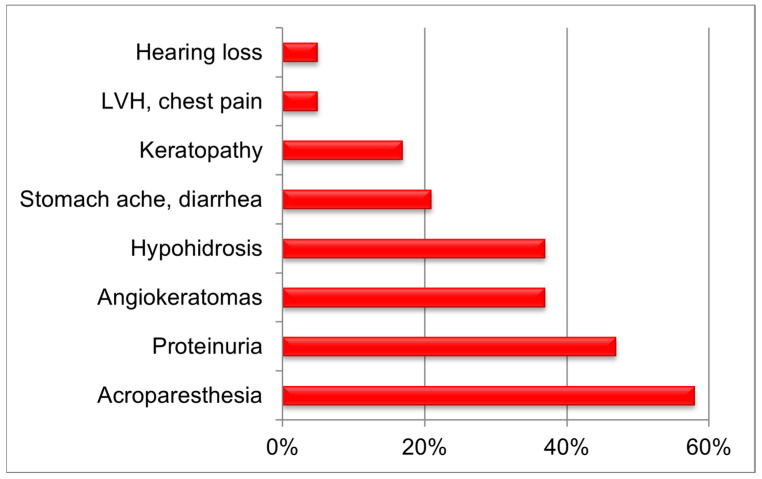
The incidence of clinical signs of FD in the examined children.

The molecular genetic features of FD development in the Russian Federation were performed by assessing 293 patients (146 men and 147 women) with causal variants of the *GLA* gene, and 102 of these patients were primary patients of screening and 191 were their relatives. All presented patients were from 133 unrelated families inhabiting 34 (39.5%) regions of the Russian Federation. The correlation calculation was performed in 114 patients with FD, because of data absence. Overall, 55 (18.7%) patients under the age of 18 years were identified, 30 boys and 25 girls, their mean age was 10 years and 6 months, and their median age was 10 years at the time of the study. At the same time, we identified five boys and one girl with a change in the reference sequence of the *GLA* gene as part of the initial screening. It is worth mentioning that the percentage of patients under 18 years of age exceeded the percentage of children (total number described) in the European register (15.0%) [31]. This can be attributed to the pediatric profile of the FSAI “National Medical Research Center of Children’s Health” of the Ministry of Health of the Russian Federation. Clinical features of FD were described in 20 children (13 boys and 7 girls) examined at the “National Medical Research Center of Children’s Health” [32] (Figure 1).

The most common manifestation of the classical FD manifested at the age of 5 to 11 years in most cases with acroparesthesia (median age was 7 years in boys and 9 years in girls) among examined children was neuropathic pain–acropareshesia (58.0%), while proteinuria was identified in 47.0% of children, angiokeratoma in 37.0%, hypohidrosis in 37.0%, stomach ache and/or diarrhea in 21.0%, keratopathy in 17.0%, left ventricular hypertrophy and/or chest pain in 5.0%, and tinnitus and/or hearing loss also in 5.0%. Interestingly, gender characteristics, age of manifestation, and early description of phenotype in children with FD showed a similar distribution of clinical signs [33]. However, our study has identified a higher incidence of proteinuria and a lower incidence of keratopathy in children with FD; this could be associated with population characteristics.

**Figure 2:**
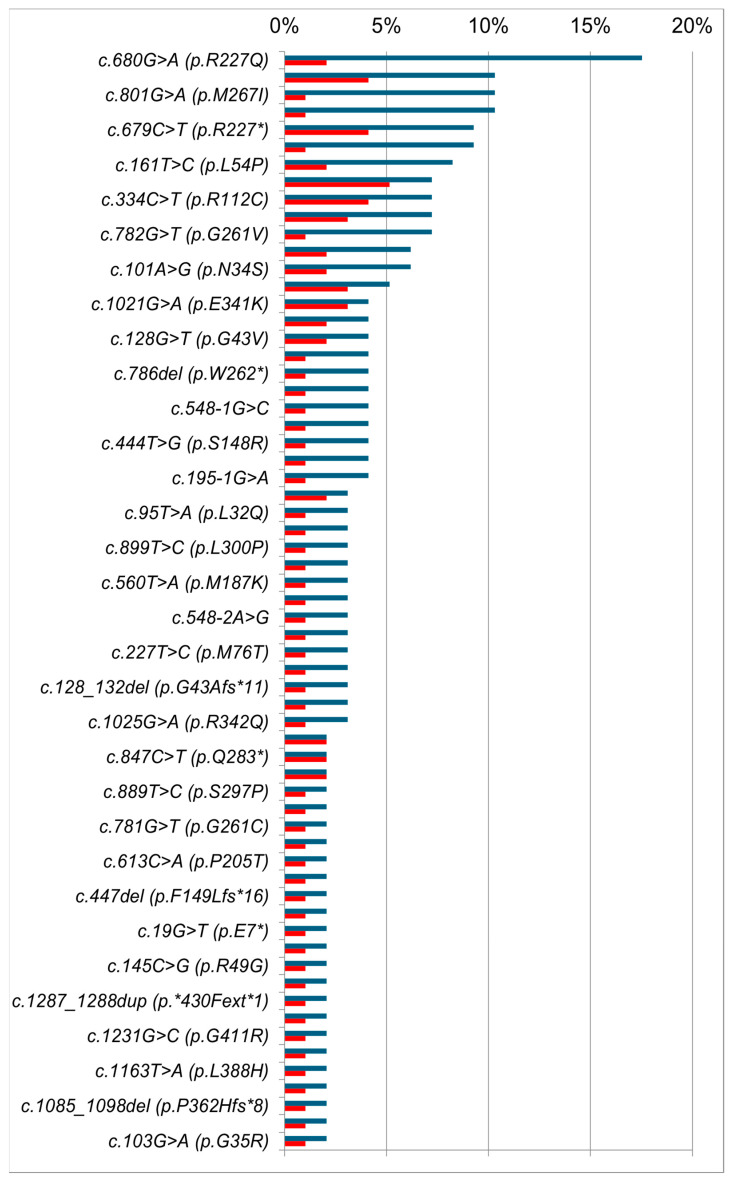
Relative frequencies and variant spectrum of the *GLA* gene variants identified among the examined Russian patients (blue columns correspond to relative frequencies of different *GLA* gene variants identified in 293 examined patients; red columns correspond to the relative frequencies of the different *GLA* gene variants identified in 133 unrelated families). Variants that occurred only once are not displayed on the graph.

The molecular genetic testing of target regions of the *GLA* gene in 293 examined patients identified 104 different variants, which amount to 7% of all variants described in the international HGMD database by the time of the study (Table A1). Relative frequencies were calculated for all identified *GLA* gene variants, and in addition, the prevalence of variants was calculated in unrelated families (Figure 2).

Most of the *GLA* gene variants (identified by us) are specific to single families, which in general is a typical feature of X-linked diseases, and FD in particular [34]. Only 17 (16.4%) *GLA* variants were identified in two or more families with FD, while each of the remaining variants was detected in members of the same family. The most common variant of the *GLA* gene among the examined families was the nonsense variant *c.658C>T*, leading to the stop codon p.R220* and was identified in five (3.8%) unrelated families and in seven (2.4%) patients, and it was previously described in patients with classical FD [35].

Other most common variants were the nonsense variants *c.644A>G* (*p.N215S*), *c.679C>T* (p.R227*), and *c.334C>T* (*p.R112C*). Each of them was identified in four (3%) families. Meanwhile, nonsense variant *c.679C>T*, which led to the stop codon p.R227* was identified in nine (3.1%) patients and was also described earlier in patients with classical FD [36].

Each of the missense variants *c.717A>G* (*p.I239M*), *c.902G>A* (*p.R301Q*), and *c.1021G>A* (*p.E341K*) were identified in three (2,25%) families, while missense variants *c.680G>A* (*p.R227Q*), *c.161T>C* (*p.L54P*), *c.128G>T* (*p.G43V*), *c.269G>A* (*p.C90Y*), and *c.101A>G* (*p.N34S*); nonsense variants *c.901C>T* (p.R301*), *c.1134T>A* (p.C378*), and *c.847C>T* (p.Q283*); and frameshift deletions *c.996_999del* (p.Q333Efs*14) and *c.718_719del* (p.K240Efs*9) were identified in two (1.5%) families. The other variants of the *GLA* gene were identified in only one family.

**Figure 3:**
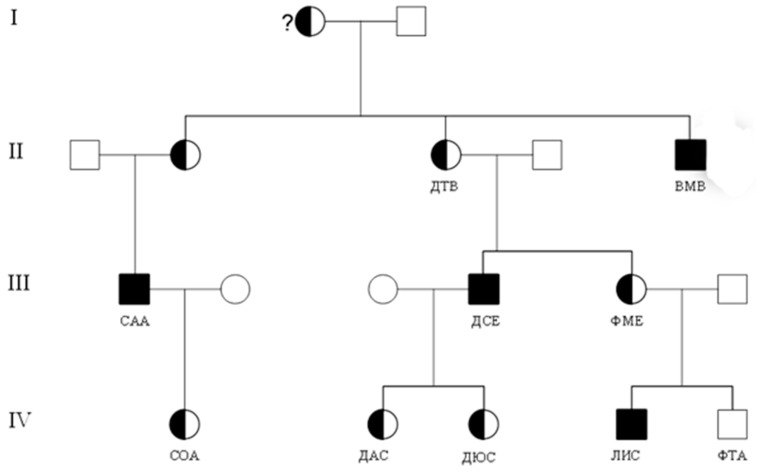
Family tree model for FD caused by variant *c.1156C>T* (p.Q386*) of the GLA gene.

The most common variant in examined patients was the missense variant *c.680G>A* (*p.R227Q*), described earlier both in patients with the classical FD [7] and in patients with the atypical cardiac form of FD [37], and it was detected in 17 (5.9%) patients. Slightly less common was the nonsense variant *c.1156C>T* (p.Q386*) that was identified in 10 (3.4%) patients from three related families living in one village located in the Nizhny Novgorod region (Figure 3). We also identified the pathogenic variant *c.644A>G* (*p.N215S*) in 10 patients from four unrelated families.

The largest number of patients with pathogenic variants of the *GLA* gene among relatives was nine; furthermore, no relatives with the same variants were identified in 44 unrelated patients.

**Figure 4:**
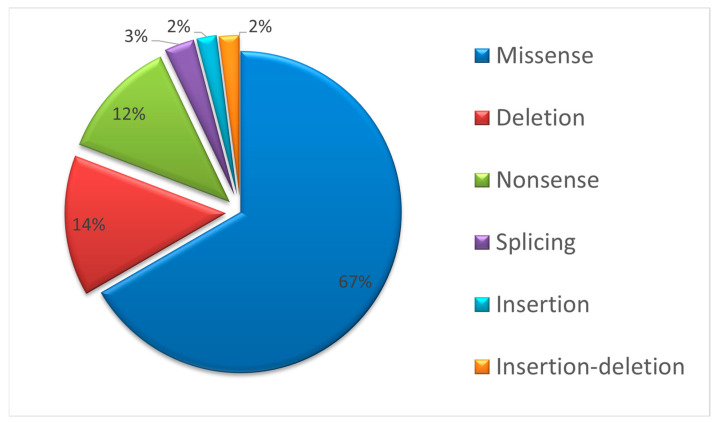
Proportions of different *GLA* variant types identified in Russian patients.

Missense variants significantly dominated among all *GLA* gene variants (70/67.3%) as they were detected in 197 (67.2%) examined patients. The details of the other identified variants are as follows: 38 (13.0%) patients had 19 (18.3%) different variants of insertions and deletions; 47 (16.0%) patients had 12 (11.5%) different variants of nonsense variants; and 11 (3.8%) patients had three (2.9%) variants leading to splice site disruption (Figure 4).

The study identified 34 (32.7%) pathogenic variants of the *GLA* gene that were not previously described in the HGMD database. All novel variants were identified in single Russian families with FD; this could confirm the global trend of FD as well as indicate trh significant variability and insufficient examination of citizens of the Russian Federation. Clinical features of FD in 14 patients with variants not described in the HGMD database are presented in Table 1.

The analysis of the identified pathogenic variants of the *GLA* gene and their link with estimated biochemical indicators facilitated the identification and description of a number of correlations. The data on age of diagnosis and lyso-Gb3 levels prior to ERT were available for 37 patients with FD out of 82 (28.2%) patients with the *GLA* gene variants that led to the synthesis of the shortened protein. These data contributed to the identification of significant differences (*p* = 0.003) in the lyso-Gb3 levels in patients with “quantitative” *GLA* variants and any other variants. Thus, no significant differences in the age of diagnosis were found between patients with “quantitative” and any other *GLA* variants (*p* = 0.933) (Table 2).

According to the data from Table 2, it is clear that the median lyso-Gb3 levels in patients with variants that prematurely terminate the encoded protein synthesis are more than three times greater than the median lyso-Gb3 levels in patients with any other *GLA* gene variants.

Moreover, the group distribution based on gender clearly shows significant differences between the genders not only in terms of the lyso-Gb3 levels (*p* < 0.001) but also in terms of the age of diagnosis (*p* = 0.032) (Table 3).

Thus, the median lyso-Gb3 levels in men are 10 times higher than in women, while the age of diagnosis in men is significantly lower than in women.

Individuals with late atypical FD with renal or cardiac manifestations have missense variants that often show a significant residual activity of α-galactosidase A (15–30% of the normal activity level in men) and low levels of lyso-Gb3 (6.3 ± 2.3 ng/mL in men), as previously described [5]. Thus, the analysis of clinical findings of carriers of variants *c.644A>G* (*p.N215S*), *c.717A>G* (*p.I239M*), and *c.758T>C* (*p.I253T*) shows the late age of diagnosis and lesions in only one of the three body systems (kidneys, heart, or central nervous system) compared to patients with pathogenic variants associated with the classical phenotype. This clearly corresponds to international data [38,39]. We also identified an intrafamilial phenotypic variability of the pathogenic variant *c.644A>G* (*p.N215S*) of the *GLA* gene: one of two brothers had developed chronic end-stage renal disease at the age of 31, while the second one sometimes had heartache with normal renal function and normal urine protein levels. This may indicate the presence of various factors influencing the development of the disease [40]. The lyso-Gb3 levels measured in males whose genome contains such variants are significantly different from the lyso-Gb3 levels measured in men with any other *GLA* gene variants (*p* ≤ 0.001) (Table 4).

This indicates the possible use of the lyso-Gb3 biomarker as the predictor of classical or atypical FD development in patients with previously undescribed genome variants [41]. With regard to the low lyso-Gb3 levels, as well as atypical disease course, in a 68-year-old patient with renal disease, a novel variant *c.895G>C* (*p.D299H*) was also referred to the mild variant of the *GLA* gene. Data from Table 5 clearly demonstrated that the age of diagnosis differs between patients groups with variants causing atypical FD phenotype and patient groups with any other variants. However, these differences were only moderately significant, while international scientists who conducted similar studies on significantly larger samples of FD patients identified significant differences both in the accumulated substrate levels and in the age of diagnosis between patient groups with classical FD and atypical FD, and could be caused by the different genome variants [42,43].

Moreover, we managed to describe variants identified in patients with early stroke: *c.723dup* (p.I242Yfs*8) (woman, 45 years old), *c.782G>T* (*p.G261V*) (woman, 18 years old; woman, 22 years old), *c.1277_1278del* (p.K426Rfs*11) (man, two strokes at the ages of 32 and 33), *c.109G>A* (*p.A37T*) (man, 49 years old), *c.1025G>A* (*p.R342Q*) (man, two strokes at the ages of 35 and 36), c.658C>T (p.R220*) (man, 29 years old), *c.844A>C* (*p.T282P*) (man, 26 years old), *c.835C>A* (*p.Q279K*) (woman, two strokes at the ages of 49 and 52), c.1134T>A (p.C378*) (man, several strokes at the ages of 26, 27, and 28), *c.612G>C* (*p.W204C*) (man, 44 years old), and *c.1156C>T* (p.Q386*) (man, 32 years old). The age of stroke in patients with *c.679C>T* (p.R227*) and *c.1197C>A* (p.W399*) variants could not be specified. There were no significant differences (*p* = 0.073) in patients with nonsense and frameshift variants (in the group of patients with FD and early stroke) in comparison to patients with any other pathogenic variants of the *GLA* gene (Table 5).

However, it was identified that men with nonsense and frameshift variants of the *GLA* gene exhibited a significant correlation (*p* = 0.005) with stroke. The odds ratio for stroke in such men compared with men with any other *GLA* gene variants was OR = 7.9 (CI 1.5–55.4) (Table 6). Women with FD and stroke did not show any significant correlation with variant types (*p* = 0.516).

Variants in patients with end-stage renal disease were also specified: *c.1228A>G* (*p.T410A*), *c.786del* (p.W262*), *c.901C>T* (p.R301*), *c.818T>C* (*p.F273S*), *c.982G>C* (*p.G328R*), *c.161T>C* (*p.L54P*), *c.427G>A* (*p.A143T*), *c.166T>A* (*p.C56S*), *c.547G>A* (*p.G183S*), *c.508G>T* (*p.D170H*), *c.679C>T* (p.R227*), *c.19G>T* (p.E7*), *c.1197G>A* (p.W399*), *c.658C>T* (p.R220*), *c.109G>A* (*p.A37T*), *c.203T>C* (*p.L68P*), *c.612G>C* (*p.W204C*), *c.644A>G* (*p.N215S*), *c.821G>A* (*.G274D*), *c.834del* (p.Q279Sfs*3), *c.895G>C* (*p.D299H*), *c.1134T>A* (p.C378*), and *c.1025G>A* (*p.R342Q*). Interestingly, there was no reliable correlation between “quantitative” variants and end-stage renal disease (*p* = 0.805) (Table 7).

The distribution of patients with ESRD based on gender showed only a low level of significance in men with nonsense and frameshift variants of the *GLA* gene (*p* = 0.096).

It should be mentioned that the measurement of lyso-Gb3 levels made it possible to identify its significantly high levels in patients with ESRD and mainly in patients with ESRD who have had at least one stroke (Table 8 and Table 9). These data indicated that patients with ESRD and history of at least one stroke had the median lyso-Gb3 levels almost double than the median lyso-Gb3 levels in patients who had developed ESRD without any stroke. This may indicate the severity of FD clinical symptoms and may be an adverse prognostic factor for stroke in patients with ESRD.

Moreover, we managed to identify significant differences in the median lyso-Gb3 level in men with FD before and during ERT (*p* < 0.001) (Table 10). This validates the findings of our international colleagues that infer that this biomarker can be used for ERT monitoring [44].

We were able to measure the lyso-Gb3 level only in one woman with FD on ERT; thus, it was not possible to process her data via the Wilcoxon test, but we were able to record a slight decrease in the lyso-Gb3 level 6 months after the therapy initiation from 9.4 ng/mL to 7.3 ng/mL.

## 4. Conclusions

In this study, we have summarized data on the spectrum and frequencies of causal variants of the *GLA* gene in 293 Russian patients from 133 unrelated families identified as a result of various selective screening programs among high-risk groups, including screening among patients with HCM using the next-generation sequencing technology as a first-tier test. Although we were unable to identify major variants of the *GLA* gene that are typical for Russian patients with FD, we have deciphered relevant genotype–phenotype correlations. Thus, we have shown for the first time that there is significant association (*p* = 0.005) of the development of stroke in men with their genome containing nonsense and frameshift variants of the *GLA* gene. Moreover, significant correlations of lyso-Gb3 levels and early stroke (*p* = 0.003) and early stroke in patients with ESRD (*p* = 0.004) were identified.

## Figures and Tables

**Table 1:** Clinical characteristics of patients with causal variants of the *GLA* gene that were not previously described.

Nucleotide Variant	Amino Acid Variant	Gender	Age of Diagnosis, Years	Lyso-Gb3, ng/mL	Keratopathy	Hypohidrosis	Acroparesthesia	Angiokeratoma	LVH, Chest Pain	Strokes	Proteinuria/Microalbuminuria	ESRD (Dialysis/Transplantation)	Hearing Loss	Stomachache/Vomiting/Diarrhea	Frequent Bronchitis
*c.203T>C*	*p.L68P*	m	52	N/A	+	+	+	–	+	–	+	+	–	+	–
*c.521G>A*	*p.C174Y*	m	18	N/A	–	–	+	–	–	–	+	–	–	–	–
*c.539_547 + 9del*	*p.L180_G183delinsC*	m	27	15.0	–	+	+	+	–	–	–	–	–	+	–
*c.804A>C*	*p.L268F*	m	19	75.9	+	+	+	–	–	–	–	–	–	+	–
*c.821G>A*	*p.G274D*	m	27	19.6	+	+	+	+	–	–	+	+	–	+	+
*c.844A>C*	*p.T282P*	m	30	17.3	+	+	+	+	–	+	+	–	+	+	–
*c.889T>C*	*p.S297P*	m	37	25.2	N/A	+	+	+	–	–	+	–	–	+	–
*c.895G>C*	*p.D299H*	m	68	9.95	N/A	N/A	N/A	N/A	N/A	N/A	+	+	–	–	–
*c.902G>T*	*p.R301L*	f	22	2.1	–	–	–	–	–	–	–	–	–	–	–
*c.981A>C*	*p.Q327H*	f	13	6.0	–	+	+	–	–	–	+	–	–	–	–
*c.1163T>A*	*p.L388H*	m	18	N/A	+	+	+	+	–	–	+	–	–	–	–
*c.949del*	p.I317Lfs*31	m	21	60.2	+	+	+	+	–	–	+	–	+	–	–
*c.1134T>A*	p.C378*	m	28	73.1	+	+	+	+	+	+	+	+	+	–	+
*c.1211G>A*	*p.R404K*	f	15	0.8	–	–	+	+	+	–	–	–	–	–	–

**Table 2:** Correlation between median lyso-Gb3 levels and the age of diagnosis of patients with “quantitative” and any other *GLA* variants.

Indicator	“Quantitative” Variants	Other Variants	*p*-Value (Mann–Whitney U Test)
Lyso-Gb3 level, ng/mL	17.9 (4.5–39.7)	5.6 (2.8–24.0)	0.003
Age of diagnosis, years	34.5 (23.5–48.0)	36.5 (20.0–48.5)	0.933

Note: lyso-Gb3 levels are represented by median values and the distribution of characteristic quartiles (25–75%).

**Table 3:** Correlation between median lyso-Gb3 levels and the age of diagnosis of patients according to gender.

Indicator	Male	Female	*p*-Value (Mann–Whitney U Test)
Lyso-Gb3 level, ng/mL	30.1 (14.0–49.9)	3.0 (1.6–4.9)	<0.001
Age of diagnosis, years	32.0 (18.0–45.0)	40.5 (25.0–56.0)	<0.001

Note: lyso-Gb3 levels are represented by median values and the distribution of characteristic quartiles (25–75%).

**Table 4:** Correlation between median lyso-Gb3 levels and the age of diagnosis in men with *c.644A>G* (*p.N215S*), *c.717A>G* (*p.I239M*), *c.758T>C* (*p.I253T*), and *c.895G>C* (*p.D299H*) variants and men with any other *GLA* gene variants.

Indicator	*c.644A>G* (*p.N215S*), *c.717A>G* (*p.I239M*), *c.758T>C* (*p.I253T*), *c.895G>C* (*p.D299H*)	Other Variants	*p*-Value (Mann–Whitney U Test)
Lyso-Gb3 level, ng/mL	5.0 (4.1–8.6)	31.1 (19.1–59.5)	<0.001
Age of diagnosis, years	41.0 (38.0–48.0)	32.0 (18.0–45.0)	0.073

Note: lyso-Gb3 levels are represented by median values and the distribution of characteristic quartiles (25–75%).

**Table 5:** Correlation between “quantitative” variants and early stroke in patients with FD.

Indicator	Number of Patients with Stroke	Number of Patients without Stroke	*p*-Value (F-Test)
Other variants	7	61	0.073
“Quantitative” variants	8	24	

**Table 6:** Correlation between “quantitative” variants of the *GLA* gene and early stroke in men with FD.

Indicator	Number of Men with Stroke	Number of Men without Stroke	*p*-Value (F-Test)
Other variants	3	39	0.005
“Quantitative” variants	7	11	

**Table 7:** Correlation between “quantitative” variants and ESRD in patients with FD.

Indicator	Number of Patients with ESRD	Number of Patients without ESRD	*p*-Value (F-Test)
Other variants	18	49	0.805
“Quantitative” variants	7	25	

**Table 8:** Correlation between lyso-Gb3 levels in the group of patients with ESRD.

Indicator	Patients with ESRD	Other Patients	*p*-Value (Mann–Whitney U Test)
Lyso-Gb3 level, ng/mL	47.8 (19.6–66.3)	17.6 (2.5–29.0)	0.003

Note: lyso-Gb3 levels are represented by median values and the distribution of characteristic quartiles (25–75%).

**Table 9:** Correlation between lyso-Gb3 levels in the group of patients with ESRD who have had at least one stroke.

Indicator	Patients with ESRD + Stroke	Other Patients	*p*-Value (Mann–Whitney U Test)
Lyso-Gb3 level, ng/mL	95.5 (73.1–101.0)	31.5 (21.0–98.0)	0.004

Note: lyso-Gb3 levels are represented by median values and the distribution of characteristic quartiles (25–75%).

**Table 10:** Calculation of statistical significance in lyso-Gb3 levels before ERT and 6 months after its initiation in Russian men with FD.

ERT	Number of Patients	Lyso-Gb3 Level, ng/mL	*p*-Value (Wilcoxon Test)
Before ERT	54	39.3 (19.1–63.4)	<0.001
During ERT	12	21.3 (10.1–35.9)	

## Data Availability

Data available on request due to privacy restrictions.

## Appendix B

**Table A1:** Distribution of nucleotide variants of *GLA* gene among 293 examined patients.

Variant	Number of Cases	Number of Families
*c.680G>A (p.R227Q)*	17	2
*c.1156C>T (p.Q386*)*	10	1
*c.644A>G (p.N215S)*	10	4
*c.801G>A (p.M267I)*	10	1
*c.550T>G (p.Y184D)*	9	1
*c.679C>T (p.R227*)*	9	4
*c.161T>C (p.L54P)*	8	2
*c.334C>T (p.R112C)*	7	4
*c.658C>T (p.R220*)*	7	5
*c.717A>G (p.I239M)*	7	3
*c.782G>T (p.G261V)*	7	1
*c.101A>G (p.N34S)*	6	2
*c.901C>T (p.R301*)*	6	2
*c.902G>A (p.R301Q)*	5	3
*c.1021G>A (p.E341K)*	4	3
*c.128G>T (p.G43V)*	4	2
*c.195-1G>A*	4	1
*c.416A>G (p.N139S)*	4	1
*c.444T>G (p.S148R)*	4	1
*c.521G>A (p.C174R)*	4	1
*c.548-1G>C*	4	1
*c.718_719del (p.K240Efs*9)*	4	2
*c.723dup (p.I242Yfs*8)*	4	1
*c.786del (p.W262*)*	4	1
*c.902G>T (p.R301L)*	4	1
*c.1025G>A (p.R342Q)*	3	1
*c.1049del (p.A350Vfs*2)*	3	1
*c.128_132del (p.G43Afs*11)*	3	1
*c.146G>C (p.R49P)*	3	1
*c.227T>C (p.M76T)*	3	1
*c.269G>A (p.C90Y)*	3	2
*c.508G>T (p.D170H)*	3	1
*c.548-2A>G*	3	1
*c.551A>G (p.Y184C)*	3	1
*c.560T>A (p.M187K)*	3	1
*c.671A>G (p.N224S)*	3	1
*c.899T>C (p.L300P)*	3	1
*c.949del (p.I317Lfs*31)*	3	1
*c.95T>A (p.L32Q)*	3	1
*c.103G>A (p.G35R)*	2	1
*c.1066C>T (p.R356W)*	2	1
*c.1085_1098del (p.P362Hfs*8)*	2	1
*c.1121_1123del (p.K374_G375delinsR)*	2	1
*c.1134T>A (p.C378*)*	2	2
*c.1163T>A (p.L388H)*	2	1
*c.1197G>A (p.W399*)*	2	1
*c.1231G>C (p.G411R)*	2	1
*c.1277_1278del (p.K426Rfs*11)*	2	1
*c.1287_1288dup (p.*430Fext*1)*	2	1
*c.143A>G (p.E48G)*	2	1
*c.145C>G (p.R49G)*	2	1
*c.166T>A (p.C56S)*	2	1
*c.19G>T (p.E7*)*	2	1
*c.442_450del (p.S148_G150del)*	2	1
*c.447del (p.F149Lfs*16)*	2	1
*c.44C>A (p.A15E)*	2	1
*c.613C>A (p.P205T)*	2	1
*c.758T>C (p.I253T)*	2	1
*c.781G>T (p.G261C)*	2	1
*c.821G>A (p.G274D)*	2	1
*c.847C>T (p.Q283*)*	2	2
*c.889T>C (p.S297P)*	2	1
*c.996_999del (p.Q333Efs*14)*	2	2
*c.1024C>T (p.R342*)*	1	1
*c.1031T>C (p.L344P)*	1	1
*c.1033_1034del (p.S345Rfs*29)*	1	1
*c.1072_1074del (p.E358del)*	1	1
*c.1091C>G (p.S364C)*	1	1
*c.109G>A (p.A37T)*	1	1
*c.1139_1149del (p.P380Hfs*15)*	1	1
*c.1211G>A (p.R404K)*	1	1
*c.1228A>G (p.T410A)*	1	1
*c.127G>A (p.G43Ser)*	1	1
*c.187T>C (p.C63R)*	1	1
*c.203T>C (p.L68P)*	1	1
*c.375del (p.H125Qfs*5)*	1	1
*c.400T>G (Y134D)*	1	1
*c.496C>G (p.L166V)*	1	1
*c.503A>G (p.K168R0*	1	1
*c.539_547 + 9del (p.L180_G183delinsC)*	1	1
*c.547G>A (p.G183S)*	1	1
*c.596T>C (p.V199A)*	1	1
*c.612G>C (p.W204C)*	1	1
*c.614C>G (p.P205R)*	1	1
*c.667T>C (p.C223R)*	1	1
*c.782del (p.G261Vfs*8)*	1	1
*c.804A>C (p.L268F)*	1	1
*c.818T>C (p.F273S)*	1	1
*c.831G>A (p.W277*)*	1	1
*c.834del (p.Q279Sfs*3)*	1	1
*c.835C>A (p.Q279K)*	1	1
*c.844A>C (p.T282P)*	1	1
*c.847C>A (p.Q283K)*	1	1
*c.859T>C (p.W287R)*	1	1
*c.869T>C (p.M290T)*	1	1
*c.895G>C (p.D299H)*	1	1
*c.897C>A (p.D299E)*	1	1
*c.908T>G (p.I303S)*	1	1
*c.920C>G (p.A307G)*	1	1
*c.946del (p.V316*)*	1	1
*c.971T>G (p.L324W)*	1	1
*c.981A>C (p.Q327H)*	1	1
*c.982G>C (p.G328R)*	1	1
*c.983G>C (p.G328A)*	1	1
